# Higher levels of person-centred care are associated with lower levels of stress of conscience in hospital and municipal care: cross-sectional findings from the PCC@Work project

**DOI:** 10.1186/s12913-025-13077-x

**Published:** 2025-07-07

**Authors:** Kristoffer Gustavsson, Andreas Fors, Cornelia van Diepen, Malin Axelsson, Monica Bertilsson, Angela Bångsbo, Gunnel Hensing, Qarin Lood

**Affiliations:** 1 Institute of Health and Care Sciences, Sahlgrenska Academy, University of Gothenburg, Gothenburg, Sweden; 2 University of Gothenburg Centre for Person-Centred Care (GPCC), Sahlgrenska Academy, University of Gothenburg, Gothenburg, Sweden; 3 Region Västra Götaland, Research, Education, Development and Innovation, Primary Health Care, Gothenburg, Sweden; 4 Erasmus School of Health Policy & Management, Erasmus University Rotterdam, Rotterdam, Netherlands; 5 Department of Care Science, Faculty of Health and Society, Malmö University, Malmö, Sweden; 6 School of Public Health and Community Medicine, Institute of Medicine, Sahlgrenska Academy, University of Gothenburg, Gothenburg, Sweden; 7 Faculty of Caring Science, Work Life and Social Welfare, Department of Work Life and Social Welfare, University of Borås, Borås, Sweden; 8 Institute of Neuroscience and Physiology, Department of Health and Rehabilitation, Sahlgrenska Academy, University of Gothenburg, Gothenburg, Sweden; 9 Administration for the elderly, nursing and care, Department of Quality and development, The City of Gothenburg, Sweden

**Keywords:** Person-centred care, Stress of conscience, Health and social care professionals, Work-related health

## Abstract

**Background:**

Stress of conscience is common in health and social care, being associated with adverse health consequences, staff turnover, and poor care quality. Person-centred care (PCC), an ethical approach to care with potential to reduce stress of conscience, has been little explored across healthcare settings. This study assesses the association between perceived PCC and stress of conscience among health and social care professionals in hospital and municipal care settings.

**Methods:**

A web survey was sent to 11,554 health and social care professionals employed in hospital and municipal care settings in western Sweden. It yielded 2123 responses, and cross-sectional analyses were performed with data from 1671 professionals. The Person-Centred Care Assessment Tool was used to measure PCC, for both the full scale (P-CAT) and its subscales “Extent of Personalising Care” (EPC) and “Organisational and Environmental Support” (OES). The Stress of Conscience Questionnaire (SCQ) was used to measure the outcome stress of conscience. Bivariate correlations and linear regressions were used to analyse the data.

**Results:**

The bivariate correlations were significant and negative for P-CAT (*r*_*s*_ = − 0.399, *p <* 0.01), EPC (*r*_*s*_ = − 0.239, *p <* 0.01), and OES (*r*_*s*_ = − 0.482, *p <* 0.01) with SCQ. When adjusted for covariates, multivariate linear regressions identified negative associations for P-CAT (*B* = − 1.16, 95% CI − 1.33, − 0.99, *p* < 0.001), EPC (*B* = − 0.8, 95% CI − 1.04, − 0.56, *p* < 0.001), and OES (*B* = − 3.14, 95% CI − 3.52, − 2.78, *p* < 0.001) with SCQ, indicating that as the scores of P-CAT and its subscales increase, the SCQ score decreases.

**Conclusions:**

Our findings revealed that hospital and municipal health and social care professionals who perceived higher levels of PCC also perceived lower levels of stress of conscience. Considering the increased focus on PCC internationally, the results are relevant, as PCC might be one possible approach to mitigate stress of conscience. More knowledge of EPC and OES in relation to stress of conscience could be important for improved and better-targeted PCC implementation efforts.

**Supplementary Information:**

The online version contains supplementary material available at 10.1186/s12913-025-13077-x.

## Background

The World Health Organization (WHO) has called for health systems to adopt a more integrated people-centred approach as a crucial component in providing high-quality care [1]. Hence, the “good quality, local healthcare” reform was developed in Sweden by the Ministry of Social Affairs, to facilitate an exhaustive organisational shift across settings towards person-centred integrated care [2]. The Organisation for Economic Co-operation and Development (OECD) highlights that health and social care professionals and their ability to provide person-centred care (PCC) are critical for health systems designed around people’s needs [3]. PCC is based on philosophical and ethical values, highlighting respect for patients’ needs and preferences. It takes a step away from seeing patients as passive care recipients and instead draws attention to their capabilities and reciprocal relationships with professionals [4].

Health and social care professionals are exposed to stressors in the workplace, found to be associated with factors such as burnout, job performance [5], and job satisfaction [6]. Jokwiro et al. [7] concluded in a scoping review that stress of conscience provides a contemporary framework in which to assess violations of personal and professional standards among health and social care professionals. They identified several studies demonstrating that a “troubled conscience” commonly occurs in situations of dissonance between professionals’ conscience and healthcare constraints [7]. Herttalampi and Feldt [8] suggested, based on findings from their longitudinal research, that professionals supported with resources to provide care in line with their ethical values could facilitate improvements in stress of conscience and retention [8].

The WHO has emphasised the need to co-produce care in health systems, in which care should be provided through collaboration between professionals and patients [1]. A qualitative study of professionals working in Australian residential aged care facilities found that a person-centred culture in the work environment was experienced as becoming incorporated into an organisational culture involving an upward spiral, which may enhance professionals’ health and well-being [9]. In addition, a qualitative systematic review from our research group found that PCC can generate feelings of job satisfaction among healthcare professionals, derived from meaningfulness and improved relationships with co-workers and patients [10]. Another systematic review by Brownie and Nancarrow [11] showed that PCC significantly affected job satisfaction and the meeting of patient needs. Ekman [12] further described how providing PCC implies a practice of ethical consideration and action by professionals striving to improve patients’ well-being [12], while Jokwiro et al. [7] proposed that spending time with patients as part of PCC could potentially mitigate stress of conscience among health and social care professionals [7].

Glasberg et al. [13] described stress of conscience as “a product of the frequency of the stressful situation and of the perceived degree of troubled conscience as rated by healthcare personnel themselves” [13]. Stress of conscience can vary depending on sociodemographic factors, such as age which has been identified as a protective factor [14] and Åhlin et al. [15] found that women experienced higher stress of conscience levels than did men. The type of profession is also relevant, as shown by Munkeby et al. [16], with registered nurses being more likely to experience stress of conscience than members of several other health and social care professions [16]. Early studies of stress of conscience, conducted in Swedish settings, have found associations, for example, with feelings of inadequacy [17, 18] and job strain [19]. Additionally, organisational changes have been found to be associated with several nursing occupations’ experiences of stress of conscience, but the association can differ depending on the nurses’ ability to address changes and the amount of support they receive from management [20]. In longitudinal research by Åhlin et al. [21], involving nursing professionals in Swedish residential care, social support from superiors displayed associations with lower levels of stress of conscience [21], and a more recent longitudinal study in a Finnish hospital district [8] found stress of conscience to be associated with burnout and turnover intentions.

In our previous research on PCC and stress of conscience, a cross-sectional pilot study in a Swedish hospital setting found an association between the Person-Centred Care Assessment Tool (P-CAT, used to measure PCC) results and stress of conscience. The association was explained by the P-CAT subscale “Organisational and Environmental Support” (OES), whereas no association was found for the subscale “Extent of Personalising Care” (EPC). In other cross-sectional studies of PCC and stress of conscience, significant associations have been identified [22, 23], whereas a multi-centre intervention study obtained no significant results [24]. However, earlier work has mainly focused on aged-care settings and nursing professionals, whereas PCC is a care approach applicable to the whole health system and all professionals working within it [25], who can experience stress of conscience in different settings [7]. Hence, the present study sought to build on earlier work by involving hospital and municipal care settings and a large sample of various health and social care professionals. The purpose was to improve our understanding of the association of PCC and the P-CAT subscales with stress of conscience, which could contribute to improved and more tailored PCC implementation. In the long run, this could improve work-related health, retention of the health and social care workforce, and care quality. The aim of the study was to assess the association of perceived PCC, measured with P-CAT and its subscales EPC and OES, with stress of conscience among health and social care professionals in hospital and municipal care settings.

The hypotheses to be tested were:

- H1: A higher total P-CAT score is associated with a lower level of stress of conscience.
- H2: A higher EPC score is associated with a lower level of stress of conscience.
- H3: A higher OES score is associated with a lower level of stress of conscience.

## Methods

### Design

This is a cross-sectional study based on baseline data from the prospective, longitudinal PCC@Work project on work-related health among health and social care professionals in hospital, municipal, and regional primary care settings [26]. Cross-sectional analyses of the baseline data contribute to the identification of associations between variables which can later be further analysed in longitudinal research. The study was conducted in line with the Declaration of Helsinki [27] and was reported according to the Strengthening the Reporting of Observational Studies in Epidemiology (STROBE) recommendations for cross-sectional studies [28] (Supplementary File 1).

### Setting and participants

Sweden has a decentralised health and social care system with 21 regional and 290 municipal authorities responsible for delivering health and social care. Regions have the main responsibility for organising and financing medical and specialised care for the population and own nearly all hospitals, whereas municipalities are generally responsible for health and social care in people’s homes when needed [29].

To obtain variation in the sample, we contacted public hospitals and municipal care providers in urban and rural areas (for logistic reasons, findings regarding regional primary care will be reported in a separate study). Information about the study and the inclusion criteria were shared at senior health and social care managers’ meetings for public hospitals and municipal care in the region. Interested managers contacted us, and email addresses of staff to receive the survey were forwarded. Two medium-sized hospitals and eight municipal care organisations– i.e., residential aged care facilities (including short-term beds), healthcare units, and rehabilitation units– agreed to participate. Health and social care professionals directly caring for and treating persons needing health and/or social care were eligible to participate.

### Data collection

A web-based survey was distributed between December 2023 and February 2024. Before distribution, the survey was pilot tested on five professionals with different professional backgrounds working in hospitals, municipal care, or primary care centres. The web-based survey was sent by e-mail to health and social care professionals in the participating organisations by a data collection company with extensive experience in implementing web-based surveys. The e-mail comprised an individual link to the survey together with information about the study. Three reminders were sent during the data collection period. The data collection company compiled the database for the analysis but had no role in analysing or presenting the findings.

**Fig. 1:**
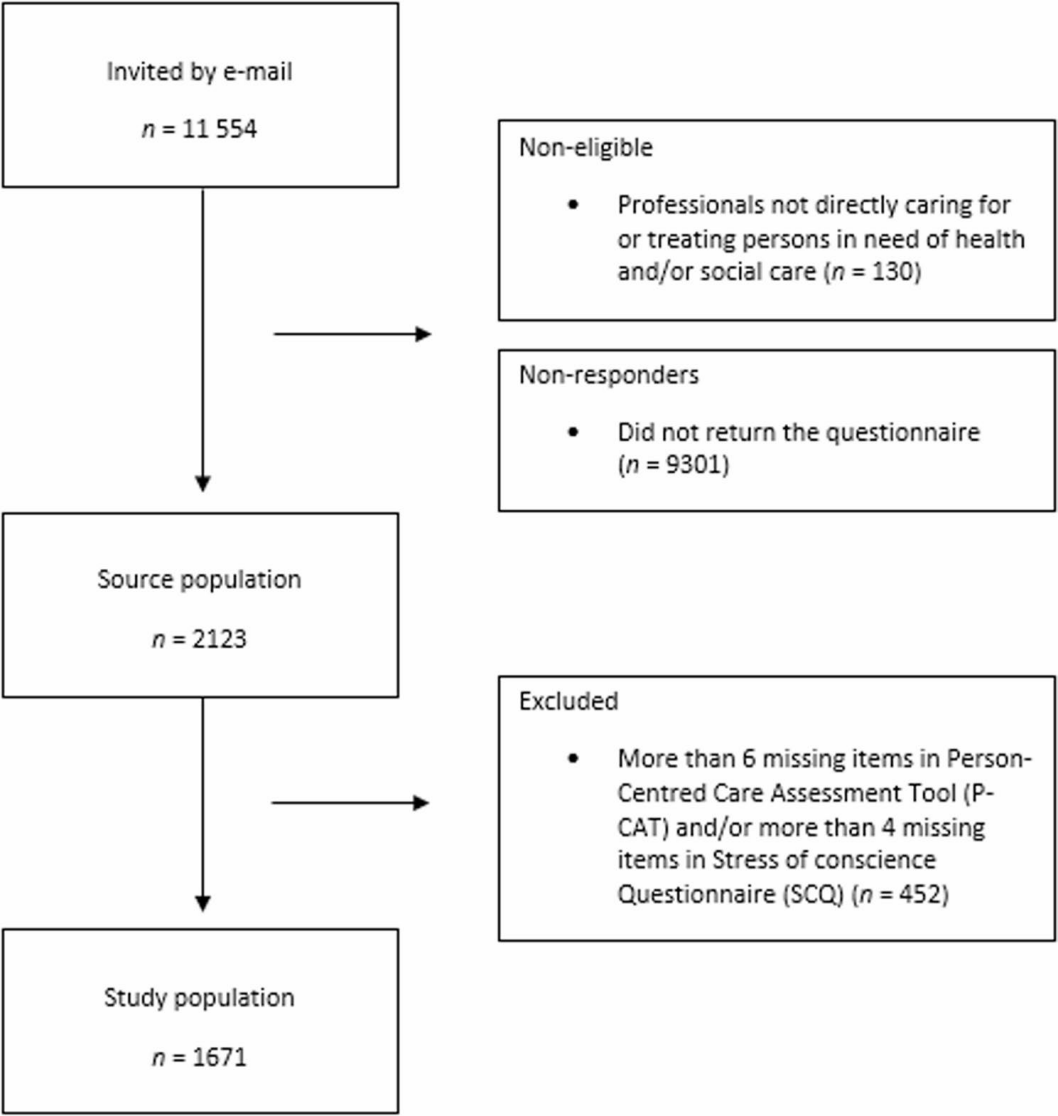
Flow chart of the inclusion process

The survey invitation was sent to 11,554 health and social care professionals, of whom 130 were ineligible because they were not health and social care professionals working directly in the care or treatment of persons needing health and/or social care. In total, 2123 professionals responded, yielding a response rate of 19%. A total of 452 surveys had more than 50% missing items for either the exposure or outcome variable and were excluded, resulting in data from 1671 professionals for analysis (Fig. 1).

### Measures

#### Person-centred care

The exposure variable was the level of perceived PCC in daily work, measured using an adapted version of P-CAT [30] comprising 13 items, similar to the original version developed by Edvardsson et al. [31]. The adapted version [30] was adjusted to suit hospital care and was further adapted in this study to suit the more diverse study population and current regulations. Item 12, phrased “Evaluation of the patient’s needs and resources is undertaken on a daily basis” in the previously adapted version [30], was changed to “Evaluation of the patient’s needs and resources is undertaken regularly” to suit the outpatient care setting. In addition, item 13 was rephrased from “We let the patient take part of the documentation from their medical record” to “We ensure that the patient has access to their care plan”, because all patients have the legal right to access their medical records [32]. The adaptations were conducted by the research group, discussed with the developer of the original P-CAT, and pilot tested along with the full survey on five professionals with varying backgrounds, working in hospitals, municipal care, and primary care centres. Both the original and adapted versions of P-CAT consist of two subscales. In the adapted versions, EPC consists of nine items (1, 2, 3, 4, 5, 6, 7, 12, and 13), and OES includes four items (8, 9, 10, and 11) (Table 1).

**Table 1:** Adapted Person-Centred Care Assessment Tool (P-CAT) items, content, and associated subscales

Item number ^a^	Content	Subscale
1	We often discuss how to give person-centred care.	EPC
2	We have formal team meetings to discuss patients’ care.	EPC
3	We listen to the patient’s story.	EPC
4	We write a care plan together with the patient.	EPC
5	The patient’s story is formally used in the care plans we use.	EPC
6	The quality of the interaction between staff and patients is more important than getting the tasks done.	EPC
7	We are free to alter work routines based on patients’ preferences.	EPC
8	I simply do not have the time to provide person-centred care.	OES
9	The environment feels chaotic.	OES
10	We have to get the work done before we can worry about a pleasant environment.	OES
11	This organisation prevents me from providing person-centred care.	OES
12	Evaluation of the patient’s needs and resources is undertaken regularly.	EPC
13	We ensure that the patient has access to their care plan.	EPC

*EPC* Extent of Personalising Care, *OES* Organisational and Environmental Support

^a^In items previously using the word “resident”, that word was replaced with “patient”; in item 10, the word “homelike” was replaced with “pleasant”; and items 12 and 13 were rephrased from the P-CAT used in the pilot study [30]

Each item has a five-point response scale ranging from 1 (“disagree completely”) to 5 (“agree completely”). There are four negatively worded items, and these were reversed before analysis. The overall P-CAT score ranges from 13 to 65, with a higher score signifying a greater level of perceived PCC. The EPC subscale ranges from 9 to 45 and the OES subscale from 4 to 20. Satisfactory internal consistency reliability was shown in this dataset, with a Cronbach’s α value of 0.84 for the total P-CAT, 0.85 for the EPC subscale, and 0.82 for the OES subscale. A validation of the P-CAT used in this study is recommended to gain a better understanding of its psychometric properties and applicability in various health and social care settings.

#### Stress of conscience

The outcome variable stress of conscience was measured using the Stress of Conscience Questionnaire (SCQ) developed by Glasberg et al. [13]. The index comprises nine A and nine B questions. The A questions are related to the frequency of a stressful situation, with the responses ranging from 0 (“Never”) to 5 (“Every day”). For the B questions, the respondent rates to what degree the situation gives them a troubled conscience using a response scale ranging from 0 (“No, not at all”) to 5 (“Yes, a very troubled conscience”). In the web-based survey, those answering 0 (“Never”) to an A question, were not shown the corresponding B question. The A-question score is multiplied by the corresponding B-question score, for a score of 0–25. The total SCQ score thus ranges from 0 to 225 [13, 18], with a higher score indicating more stress of conscience.

The psychometric properties of the index have been demonstrated to be satisfactory and reliable for use in the Swedish health and social care context (overall scale, Cronbach’s α = 0.83) [13, 18]. The index has also been demonstrated to be reliable for Swedish and Finnish primary and municipal care [33, 34]. The Cronbach’s α for the total SCQ score in this dataset was 0.86.

#### Covariates

The covariates adjusted for in the regression models were sex, age, profession, and intent to leave. The intent to leave covariate was measured using one question from the Copenhagen Psychosocial Questionnaire (COPSOQ III): “How often do you consider looking for work elsewhere?”, with the response alternatives being “Never/hardly ever”, “Seldom”, “Sometimes”, “Often”, and “Always” [35]. Dummy variables were created for each profession (i.e., assistant nurse, registered nurse and specialist nurse, physician, occupational therapist, physiotherapist, and other professions) and for each response alternative of the intent to leave covariate.

### Statistical analyses

IBM SPSS Statistics version 29 was used to analyse the data [36]. *P*-values below 0.05 were considered statistically significant. The data distribution can be considered approximately normal when skewness is between − 1 and + 1 [37]. In the present study, the skewness of the exposure variable P-CAT was–0.429 and of SCQ was 0.996. Thus, no value was outside the range of an approximately normal distribution [37].

The half-rule principle was applied, so those with missing data but fewer than 50% missing items in P-CAT (maximum six missing) and SCQ (maximum four missing) were imputed. Missing data for P-CAT were 0.6% of values and 4.7% of cases, and for SCQ, 4% of values and 24.2% of cases. Since no systematic reasons for the missing data were identified and the proportion of missing values was low, individual mean imputation was deemed a suitable imputation method [38], and was executed using the scale mean of the variable for each respondent [39].

As a first step, descriptive statistics were used to explore frequencies, distributions, means, and standard deviations (SD) for P-CAT and SCQ. Demographic data were stratified to show the means and SDs of the exposure and outcome variables in different groups to provide an overview of the sample and context for interpreting the results. Second, bivariate correlation analyses of the exposure variable P-CAT and its subscales EPC and OES and of the outcome variable SCQ were conducted using Spearman’s rank correlation coefficient (*r*_*s*_) to evaluate the strength and direction of the correlation between the variables. The bivariate correlations were calculated within stratified groups, categorised by setting and professional group, to explore potential differences within these groups. The bivariate correlation analyses were executed using bias-corrected accelerated 95% bootstrapped confidence intervals with 5000 resamples for robust estimates [40].

Third, assumptions for linear regression were tested, showing linearity, normal distribution of residuals, no univariate outliers, and Cook’s distance values less than 1 (highest value was 0.011), indicating no influence of multivariate outliers. Multicollinearity was rejected as the variance inflation factors (VIFs) were under 10 (1.1–1.6) and the tolerance values were above 0.1 (0.64–0.94) [41]. Visualisation of scatter plots, along with Breusch-Pagan and Koenker tests, did not support the assumption of homoscedasticity of residuals. To correct for the heteroscedasticity, bias-corrected accelerated 95% wild bootstrapped confidence intervals from 5000 resamples were generated for linear regression analyses [42].

Fourth, multivariate linear regression analyses were conducted without stratifying for the setting because the associations in the bivariate correlation analyses of P-CAT and SCQ were similar in hospital and municipal care. Regression analyses were executed without covariates, and in three models with adjustments for covariates. Sensitivity analyses were conducted for the bivariate correlations and the regression analyses, with and without imputed data, indicating no significant differences.

## Results

Participant characteristics are presented in Table 2. The 1671 participants included in the analyses were aged 20–76 years, with a mean age of 48 years (SD ± 11.6, median 49 years), and most were women (*n* = 1356, 81%). Most worked in hospitals (*n* = 1026, 61%). The professions represented were predominantly assistant nurses (*n* = 683, 41%), registered and specialist nurses (*n* = 492, 30%), and physicians (*n* = 155, 9%). Most had over ten years of experience working in health and/or social care (*n* = 1089, 66%). For the total sample, the mean P-CAT score was 47 (SD ± 9.3) and the mean SCQ score was 38.1 (SD ± 32.9). The scores varied among professions, for example, with assistant nurses reporting mean scores of 49.2 (SD ± 8.7) for P-CAT and 39.2 (SD ± 35.1) for SCQ, and physicians reporting mean scores of 42.5 (SD ± 9.1) for P-CAT and 47.9 (SD ± 33.6) for SCQ. More detailed information is available in Table 2.

**Table 2:** Participants’ demographic characteristics and self-reported levels of person-centred care and stress of conscience

	Total sample			Hospital		Municipal care	
Variables	*n* (%)^a^	*P*-CAT, mean ± SD	SCQ, mean ± SD^b^	*n* (%)	SCQ, mean ± SD	*n* (%)	SCQ, mean ± SD
	1671	47 ± 9.3	38.1 **±** 32.9	1026	39.5 **±** 32.8	645	36.1 **±** 32.9
Gender							
Women	1356 (81.3)	47.4 ± 9.3	39.6 **±** 32.9	807 (78.8)	41.1 **±** 32.5	549 (85.2)	37.3 ± 33.1
Men	307 (18.4)	45.6 ± 9.3	31.6 **±** 32.5	215 (21)	33.8 **±** 33.6	92 (14.3)	26.4 ± 29.3
Other	5 (0.3)	42.8 ± 9.5	40.2 **±** 26.7	2 (0.2)	32.5 **±** 33.2	3 (0.5)	45.3 ± 27.8
Mean age	48 ± 11.6 (range: 20–76 years)			47 ± 11.6 (range: 20–76 years)		49 ± 11.7 (range: 21–74 years)	
Years of experience working in health and social care							
< 1 year	23 (1.4)	46.9 ± 8.3	45.7 ± 34	11 (1.1)	60.3 ± 35.1	12 (1.9)	32.3 ± 28
1–2 years	80 (4.8)	48.8 ± 7.6	38.4 ± 33.6	49 (4.8)	42.9 ± 35.3	31 (4.9)	31.2 ± 29.9
3–5 years	189 (11.4)	46.7 ± 9.3	39.8 ± 33.5	103 (10.1)	43.2 ± 34.5	86 (13.5)	35.7 ± 31.9
6–10 years	279 (6.8)	46.1 ± 8.6	39.6 ± 32.2	170 (16.7)	45.2 ± 33.5	109 (17.1)	30.8 ± 27.9
> 10 years	1089 (65.6)	47.2 ± 9.5	37.2 ± 32.9	688 (67.4)	36.9 ± 32	401 (62.8)	37.5 ± 34.4
Profession	1670						
Assistant nurse	683 (40.9)	49.2 ± 8.7	39.2 ± 35.1	281 (27.4)	38.2 **±** 33.9	402 (62.3)	39.7 ± 35.8
Registered nurse (including specialist nurses)	492 (29.5)	44.8 ± 9.4	38.5 ± 32.3	396 (38.6)	40.5 **±** 33.4	96 (14.9)	30.1 ± 26
Physician	155 (9.3)	42.5 ± 9.1	47.9 ± 33.6	155 (15.1)	47.9 **±** 33.6	-	-
Occupational therapist	94 (5.6)	50.5 ± 7.5	27 ± 25.3	31 (3)	30.5 **±** 27.6	63 (9.8)	25.2 ± 24.1
Physiotherapist	71 (4.3)	48.6 ± 8.3	36.9 ± 26.3	20 (2)	36.3 **±** 22.4	51 (7.9)	37.1 ± 27.9
Other professions (e.g., dietician, psychologist, and speech therapist)	175 (10.5)	46.3 ± 9.2	31 ± 28	142 (13.9)	32.8 ± 28.6	33 (5.1)	22.9 ± 23.5
Intent to leave (how often looking for a new job)	1587						
Never/hardly ever	430 (27.1)	51 ± 8.3	20.3 ± 21	266 (27)	20.3 **±** 19.4	164 (27.2)	20.2 ± 23.5
Seldom	346 (21.8)	48.2 ± 8.5	29.6 ± 25.9	226 (22.9)	32.6 **±** 26.4	120 (19.9)	24 ± 23.9
Sometimes	413 (26)	45.6 ± 8.6	44.2 ± 31.1	269 (27.3)	47.4 ± 32.4	144 (23.9)	38.4 ± 27.7
Often	291 (18.3)	43.6 ± 9.4	57.6 ± 35.7	170 (17.3)	59.2 ± 36	121 (20.1)	55.3 ± 34.8
Always	107 (6.7)	41.1 ± 10.9	65.3 ± 40.9	54 (5.5)	65.6 ± 38.9	53 (8.8)	64.5 ± 43.5

*P-CAT* Person-Centred Care Assessment Tool, *SCQ* Stress of Conscience Questionnaire, *SD* Standard deviation

^a^Numbers do not always add up to 1671 due to internal attrition

^b^Despite the positive skew in the stress of conscience variable, the mean and standard deviation were reported for consistency with earlier research

### Correlations

Table 3 reports the bivariate correlations between the exposure variable P-CAT, as well as its subscales EPC and OES, and the outcome SCQ. P-CAT was negatively associated with SCQ in the total sample (*r*_*s*_ = − 0.399, *p <* 0.01), and in the hospital (*r*_*s*_ = − 0.412, *p <* 0.01) and municipal care (*r*_*s*_ = − 0.384, *p <* 0.01) settings, meaning that those reporting higher P-CAT scores also reported lower SCQ scores. For the subscales, the associations with SCQ were stronger for OES (*r*_*s*_ = − 0.482, *p <* 0.01) than for EPC (*r*_*s*_ = − 0.239, *p <* 0.01) in the total sample, and similar trends were seen for each setting. Statistically significant negative associations (*p <* 0.05) between P-CAT and SCQ were observed in all professions, with the strongest associations being observed for the OES subscale. For the EPC subscale, there were significant negative associations across all professions except for occupational therapists and physiotherapists (Table 3).

**Table 3:** Bivariate spearman’s rank correlation coefficient (*r*_*s*_) with 95% bias-corrected accelerated bootstrapped confidence intervals

	*P*-CAT and SCQ (*r*_s_)	EPC and SCQ (*r*_s_)	OES and SCQ (*r*_s_)
Total (*n* = 1671)	–0.399** (–0.439;–0.358)	–0.239** (–0.285;–0.195)	–0.482** (–0.522;–0.441)
Setting			
Hospital (*n* = 1026)	–0.412** (–0.465;–0.358)	–0.246** (–0.305;–0.186)	–0.515** (–0.566;–0.464)
Municipal care (*n* = 645)	–0.384** (–0.452;–0.316)	–0.221** (–0.301;–0.142)	–0.428** (–0.495;–0.358)
Profession			
Assistant nurse (*n* = 683)	–0.426** (–0.488;–0.360)	–0.273** (–0.343;–0.202)	–0.462** (–0.526;–0.391)
Registered nurse and specialist nurse (*n* = 492)	–0.407** (–0.486;–0.324)	–0.239** (–0.325;–0.151)	–0.526** (–0.596;–0.450)
Physician (*n* = 155)	–0.320** (–0.458;–0.169)	–0.163* (–0.312;–0.002)	–0.466** (–0.593;–0.325)
Occupational therapist (*n* = 94)	–0.254* (–0.427;–0.062)	–0.141 (–0.328; 0.057)	–0.300** (–0.480;–0.091)
Physiotherapist (*n* = 71)	–0.312** (–0.552;–0.031)	–0.192 (–0.436; 0.086)	–0.388** (–0.610;–0.131)
Other (*n* = 175)	–0.396** (–0.522;–0.260)	–0.254** (–0.394;–0.102)	–0.466** (–0.579;–0.338)

*P-CAT* Person-Centred Care Assessment Tool, *EPC* Extent of Personalising Care (P-CAT subscale), *OES* Organisational and Environmental Support (P-CAT subscale), *SCQ* Stress of Conscience Questionnaire

*p < 0.05; **p < 0.01 (two-tailed)

### Multivariate linear regression analysis

Table 4 presents the regression analyses using the exposure variable P-CAT, its subscales EPC and OES, covariates, and the outcome variable SCQ, which were conducted to assess the impact and strength of the associations. The unstandardised beta coefficients (*B*) for P-CAT were approximately similar without adjustment in the crude regression (*B* = − 1.57, 95% CI − 1.72, − 1.43, *p* < 0.001), when adjusted for sex and age in Model I (*B* = − 1.55, 95% CI − 1.7, − 1.4, *p* < 0.001), and when adjusted for sex, age, and profession in Model II (*B* = − 1.58, 95% CI − 1.73, − 1.42, *p* < 0.001). A decrease was seen in Model III (*B =* − 1.16, 95% CI − 1.33, − 0.99, *p* < 0.001) when adjusting for both previous models along with intent to leave. The unstandardised beta (*B*) for P-CAT subscales with SCQ, when adjusted for all covariates in the final model, was stronger for the OES subscale (*B* = − 3.14, 95% CI − 3.52, − 2.78, *p* < 0.001) than the EPC subscale (*B* = − 0.8, 95% CI − 1.04, − 0.56, *p* < 0.001). Statistical significance with a negative unstandardised beta coefficient (*B*) means that as the level of P-CAT and its subscales EPC and OES increases, the SCQ level decreases when adjusted for covariates. Hence, the three null hypotheses can be rejected.

The final P-CAT model explained 34% of the variance in SCQ (adjusted *R*^2^ = 0.34). For the final models of the subscales, the OES subscale explained 38% (adjusted *R*^2^ = 0.38) and the EPC subscale explained 28% (adjusted *R*^2^ = 0.28) of the variance in SCQ.

**Table 4:** Multivariate linear regression analysis with wild bootstrapped bias-corrected accelerated 95% confidence intervals

	Crude				Model I^a^				Model II^b^				Model III^c^			
	***B***	**95% CI**	**SE**	***Adj. R*** ^***2***^	***B***	**95% CI**	**SE**	***Adj. R*** ^***2***^	***B***	**95% CI**	**SE**	***Adj. R*** ^***2***^	***B***	**95% CI**	**SE**	***Adj. R*** ^***2***^
P-CAT	–1.57**	(–1.72; − 1.43)	0.076	0.19	–1.55**	(–1.7; − 1.4)	0.077	0.23	–1.58**	(–1.73; − 1.42)	0.081	0.24	–1.16**	(–1.33; − 0.99)	0.085	0.34
EPC	–1.2**	(–1.42; − 0.97)	0.113	0.06	–1.17**	(–1.38; − 0.94)	0.114	0.1	–1.25**	(–1.49; − 1)	0.123	0.13	–0.8**	(–1.04; − 0.56)	0.124	0.28
OES	–4.13**	(–4.47; − 3.83)	0.170	0.29	–4.05**	(–4.39; − 3.73)	0.171	0.31	–3.98**	(–4.32; − 3.66)	0.173	0.32	–3.14**	(–3.52; − 2.78)	0.193	0.38
Dependent variable: SCQ																

*P-CAT* Person-Centred Care Assessment Tool, *EPC* Extent of Personalising Care (P-CAT subscale), *OES* Organisational and Environmental Support (P-CAT subscale), *SCQ* Stress of Conscience Questionnaire

**p < 0.001 (two-tailed)

^a^Model I: Adjusted for sex and age

^b^Model II: Adjusted for Model I and profession (i.e., assistant nurse, registered nurse and specialist nurse, physician, occupational therapist, physiotherapist and other professions)

^c^Model III: Adjusted for previous models and intent to leave

## Discussion

In this study, we aimed to test three hypotheses regarding the association of perceived PCC, using P-CAT and its subscales EPC and OES, with stress of conscience using SCQ among health and social care professionals in hospital and municipal care settings. Support was found for all three hypotheses, indicating that health and social care professionals who reported higher scores for P-CAT and its subscales EPC and OES reported lower SCQ scores.

The association found between higher total P-CAT scores and lower SCQ scores supports H1 and corresponds well with cross-sectional studies by Backman et al. [23] and Sjögren et al. [22], who found that PCC was significantly related to lower levels of stress of conscience among nursing staff in Swedish residential aged care. Those studies used the same instruments as used here, although the adapted version of P-CAT was used in this study. This is also consistent with longitudinal research by Herttalampi and Feldt [8], showing that professionals who provide care fulfilling their intrinsic moral values perceive lower levels of stress of conscience [8]. Glasberg et al. [13] pointed out that the inability to meet patient needs could give rise to stress of conscience [13]. Our findings thus suggest the importance of hospital and municipal care professionals providing care coherent with personal ideals of quality care and care that meets patient needs, as factors that might reduce stress of conscience.

Additionally, we found support for H2, meaning that the EPC subscale revealed a significant association with lower SCQ scores. This is in contrast to a cross-sectional pilot study conducted by our research group in Sweden [30], which included hospital staff and found no association between EPC and stress of conscience [30]. A plausible explanation for this could be that the larger sample size in this study enabled the detection of significant associations. Epstein et al. [43] argued that a workplace where professionals strive to put patients first and promote their autonomy embodies an approach to care that can fulfil intrinsic moral values [43]. Informed by the findings obtained in their review, Jokwiro et al. [7] proposed that time spent with patients providing PCC could contribute to reduced stress of conscience. The significant association between EPC and lower SCQ scores suggests that considering the extent to which care is personalised in the workplace, along with the implementation of more PCC, might help mitigate health and social care professionals’ stress of conscience in hospital and municipal care.

Support was also found for H3, with a strong association between higher levels in the OES subscale and lower SCQ scores. This is consistent with Heikkilä et al. [20], who found in their cross-sectional study that management plays a crucial role in helping nurses manage stress of conscience during ongoing organisational change. By providing support and necessary resources, such as sufficient staffing, management can enable staff to provide care in line with their values [20]. Results of a cross-sectional study by Glasberg et al. [17] in a Swedish hospital district revealed that lack of managerial support was associated with stress of conscience [17], and longitudinal research by Åhlin et al. [21] found that social support from a superior could buffer nurses’ perceived stress of conscience [21]. Organisational factors, such as staffing and managerial support, should thus be emphasised for improved PCC implementations that could reduce professionals’ levels of stress of conscience. Moreover, Brownie and Nancarrow [11] found in their systematic review that organisational culture changes that involve applying PCC in practice can enhance the possibility of staff meeting patient needs [11], which in turn may contribute to lower levels stress of conscience.

In a qualitative systematic review conducted by our group [10], we found that job satisfaction could be experienced from improved relations with patients and colleagues and team collaboration when providing PCC. Additionally, the longitudinal study by Åhlin et al. [21] found a significant association between conflicts among colleagues and increased stress of conscience. In cross-sectional research, Glasberg et al. [44] suggested that staff should pay attention to their own and colleagues’ experiences of situations troubling the conscience. Viewed in relation to our findings, this suggests that a person-centred and supportive work environment created by both colleagues and superiors might serve as a factor protecting against stress of conscience. Our findings are, however, in contrast to those of a multi-centre intervention study [24] conducted in Australian, Norwegian, and Swedish residential aged care facilities that found no significant effects of a person-centred intervention on stress of conscience levels. Those authors discussed management and leadership factors that might have negatively affected the PCC implementation intervention [24]. An interview study by Moore et al. [45] revealed perceptions that organisational structures and leadership were vital factors for PCC implementation [45]. Similarly, Backman et al. [46] suggest in their cross-sectional study of Swedish residential aged care facilities that managers and leaders play a crucial role in implementing PCC by supporting staff’s knowledge and professional development, which should be considered in leadership training and development initiatives [46]. Additionally, a recent person-centred development programme for nurse leaders found that participants reported acquiring new skills while also creating a supportive organisational environment with safe spaces for the team to discuss and grow [47]. Above the first-line managers, Birken et al. [48] also point towards the importance of the senior management. They found in their mixed-methods study in the United States that when senior management emphasised the prioritisation of an implementation to first-line managers, and provided support through policies and resources such as staffing, training, and funding, it enhanced the commitment of first-line managers to execute the implementation [48]. Thus, considering the ongoing extensive organisational shift towards person-centred integrated care in Sweden [2], this study highlights a strong association between OES and lower SCQ scores. It suggests that managerial support, a positive psychosocial work environment, and efforts to provide organisational resources, such as sufficient staffing and training may improve PCC implementation and reduce stress of conscience among health and social care professionals in hospital and municipal care settings.

### Strengths and limitations

Due to the cross-sectional study design, no causal inferences can be drawn [49]. The inclusion of professionals from different settings could potentially affect the opportunity to better understand associations, as these might not be equally transferrable from one setting to another. On the other hand, the stratified bivariate correlations were similar between the different settings, and the instruments used were developed for use in both settings. Moreover, the study design allowed a larger sample size in the linear regression analyses and thus provided more statistical power, reducing the risk of type II error, i.e., failing to detect an association that is present [50].

Other strengths were that the number of missing values was low in both P-CAT (0.6%) and SCQ (4%), and that the original P-CAT and the SCQ have been validated for use in a Swedish context [13, 18, 31]. Apart from individual mean imputation, multiple imputation could also have been executed in the present study [38]. However, individual mean imputation was chosen to align with previous studies that applied it with the P-CAT and SCQ [23, 46]. In this study, the internal consistency reliability was evaluated using the Cronbach’s α values for the adapted P-CAT, with its subscales, and for SCQ, which ranged from 0.8 to 0.86. This is above the cut-off for what is widely considered an acceptable Cronbach’s α value of 0.7 [51].

The survey had a low response rate (19%), an ongoing challenge for health and social care research in Europe, where low response rates are common [52]. Reminders can increase response rates [53], so three reminders were sent. However, e-mail is not a prioritised work tool for several professions that received the survey, and we cannot with certainty discern the number of people who opened the e-mail. How many considered to participate or not is therefore difficult to ascertain, and a more accurate response rate would have been desirable. Demographics such as age and sex distribution within the professions most represented were similar to those in the regional hospitals [54], strengthening the representativeness. Information on the age and sex distribution was unavailable for health and social care professionals in the specific municipalities studied here, but it was similar to the overall demographics observed among professionals across Sweden’s municipalities [55].

Several potential biases should be considered in studies with low response rates. For example, self-selection bias could be present as health and social care professionals with either higher or lower levels of stress of conscience may be less likely to participate in surveys. This could lead to both an underestimation and an overestimation of stress of conscience levels. Moreover, measures were self-reported, which can increase the risk of socially desirable answers [56] and lead to response bias from overestimating the level of PCC. The risk of socially desirable answers was minimised by using instruments with gradual response scales and by informing respondents that individual responses would be handled confidentially to prevent unauthorised access and that no individual results would be presented.

Overadjustment bias [57] might be present in the final regression model when intent to leave was entered, as a notable decrease in the unstandardised beta and increase in the adjusted *R*^2^ was observed, which could cause underestimation of the association between PCC and stress of conscience. Another possible explanation for this could be a mediation effect, where a mediating variable conveys some of the effect of the independent variable on the dependent variable [58]. Although the intent to leave variable was used as a covariate in the final regression model, its potential role as a mediator in the relationship between PCC and stress of conscience cannot be ruled out and should be further explored. Moreover, the potential for unmeasured confounding variables should be considered in observational research [59]. Resilience might be an unmeasured confounder in this study, as it was mentioned as a factor that could facilitate PCC by participants in an implementation program evaluation report by McCormack et al. [60]. Moreover, cross-sectional research by Glasberg et al. [17] found lower resilience to be associated with stress of conscience [17], which could imply overestimation of PCC in this study. Lastly, reverse causation cannot be ruled out [61]. No or low stress of conscience might affect the perception of PCC levels at a workplace as well as patterns of questionnaire responses, presumably in the direction of a higher perceived levels.

## Conclusions

In this study in Swedish hospitals and municipal care settings we found that health and social care professionals who perceived higher levels of PCC also perceived lower levels of stress of conscience. We found a stronger association with the OES subscale than with the EPC subscale, pointing towards organisational and management issues as crucial. Considering the enhanced attention internationally to adopting more PCC, our findings are important, supporting this approach as one possible solution to reduce stress of conscience and potentially improve retention in the long run. Enhancing our understanding of the relationship of EPC and OES with stress of conscience could be valuable for improved and better-targeted PCC implementations. Nevertheless, future studies are needed in different contexts, longitudinally and with interventional designs, for more robust conclusions regarding causality and for evidence of the effects of PCC on stress of conscience among professionals in different health and social care settings.

## Supplementary Information

Supplementary Material 1.

## Data Availability

The datasets generated and analysed as part of this study are not publicly available. Data used and/or analysed in this study can be provided in de-identified form by the corresponding author upon reasonable request. All data are covered by the Swedish Public Access to Information and Secrecy Act (Offentlighets- och sekretesslagen), and a confidentiality assessment (sekretessprövning) will be performed for each individual request. Permission from the Institute of Health and Care Sciences, University of Gothenburg, must be obtained before data can be accessed. Data will be stored for 10 years after publication at the University of Gothenburg, Sweden.

## Abbreviations

WHO: World Health Organization
PCC: Person-centred care
P-CAT: Person-Centred Care Assessment Tool
EPC: Extent of Personalising Care [subscale]
OES: Organisational and Environmental Support [subscale]
SCQ: Stress of Conscience Questionnaire
STROBE: Strengthening the Reporting of Observational Studies in Epidemiology
COPSOQ III: Copenhagen Psychosocial Questionnaire
VIF: Variance inflation factor
